# *COL1A1/2* Pathogenic Variants and Phenotype Characteristics in Ukrainian Osteogenesis Imperfecta Patients

**DOI:** 10.3389/fgene.2019.00722

**Published:** 2019-08-09

**Authors:** Lidiia Zhytnik, Katre Maasalu, Andrey Pashenko, Sergey Khmyzov, Ene Reimann, Ele Prans, Sulev Kõks, Aare Märtson

**Affiliations:** ^1^Department of Traumatology and Orthopedics, University of Tartu, Tartu, Estonia; ^2^Clinic of Traumatology and Orthopedics, Tartu University Hospital, Tartu, Estonia; ^3^Department of Pediatric Orthopedics, Sytenko Institute of Spine and Joint Pathology, AMS Ukraine, Kharkiv, Ukraine; ^4^Centre of Translational Medicine, University of Tartu, Tartu, Estonia; ^5^Department of Pathophysiology, University of Tartu, Tartu, Estonia; ^6^Perron Institute for Neurological and Translational Science, QEII Medical Centre, Nedlands, WA, Australia

**Keywords:** osteogenesis imperfecta, collagen I, *COL1A1*, *COL1A2*, Sanger sequencing, bone disorder

## Abstract

Osteogenesis imperfecta (OI) is a hereditary bone disorder caused by defects of type I collagen. Although up to 90% of patients harbor pathogenic variants in the *COL1A1/2* gene, which codes for collagen α1/2 chains, the spectrum of OI genotypes may differ between populations, and there is academic controversy around OI genotype-phenotype correlations. In the current study, 94 Ukrainian OI families were interviewed. Clinical and genealogical information was collected from patients in spoken form, and their phenotypes were described. To identify the spectrum of collagen I pathogenic variants, *COL1A1/2* mutational analysis with Sanger sequencing was performed on the youngest affected individual of every family. Of the 143 patients investigated, 67 (46.85%) had type I OI, 24 (16.78%) had type III, 49 (34.27%) had type IV, and III (2.10%) had type V. The mean number of fractures suffered per patient per year was 1.32 ± 2.88 (type I 0.50 ± 0.43; type III 3.51 ± 6.18; type IV 1.44 ± 1.77; and type 5 0.77 ± 0.23). 87.23% of patients had skeletal deformations of different severity. Blue sclera, dentinogenesis imperfecta, and hearing loss were present in 87%, 55%, and 22% of patients, respectively. *COL1A1/2* pathogenic variants were harbored by 60 patients (63.83%). 27 pathogenic variants are described herein for the first time. The majority of the pathogenic variants were located in the *COL1A1* gene (76.19%). Half (49.21%) of the pathogenic variants were represented by structural variants. OI phenotype severity was highly correlated with type of collagen I defect. The current article presents an analysis of the clinical manifestations and *COL1A1/2* mutational spectrum of 94 Ukrainian OI families with 27 novel *COL1A1/2* pathogenic variants. It is hoped that this data and its analysis will contribute toward the increased understanding of the phenotype development and genetics of the disorder.

## Introduction

Osteogenesis imperfecta (OI) is a group of rare congenital disorders of the connective tissue, also known as a brittle bone disease. Up to 90% of OI is caused by collagen type I structural (i.e. qualitative) or haploinsufficiency (i.e. quantitative) defects (Shapiro, 2014). OI patients suffer from low bone mass, which results in pathological fractures and skeletal deformities. According to the severity of a collagen defect, patients typically develop bowing of long bones, rib cage deformations, scoliosis and kyphosis, triangular shape of the head, and short stature. Being the most abundant structural protein in the body, collagen type I is also altered in other tissues, causing dentinogenesis imperfecta (DI), blue sclera, muscle weakness, ligamentous laxity, easy bruising, cardiac valve and pulmonary abnormalities, and conductive or sensory hearing loss (Marini et al., 2007; Marini et al., 2017).

OI is one of the most common skeletal dysplasias among orphan diseases. Its prevalence is estimated to be 1/20,000, although this may be affected by OI type and diagnostic practice, as many mild OI cases remain underdiagnosed (Byers and Steiner, 1992). OI phenotypes range from mild osteopenia to severe perinatal lethal forms (Kocher and Shapiro; Roughley et al., 2003; Sillence et al., 1979). The clinical classification distinguishes five main OI types (Sillence et al., 1979). The four classical Sillence OI types are: type I (mild non-deforming OI with blue sclera); type II (perinatal lethal); type III (severe progressive deforming); and type IV (moderate varied OI). Type V OI involves the ossification of interosseous membranes (Glorieux et al., 2000; Amor et al., 2011; Cho et al., 2012; Semler et al., 2012).

Regardless of the various genetic causes of the remaining 10% of OI cases, their clinical manifestations usually coincide with the phenotypes of individuals with *COL1A1/2* pathogenic variants. These OI forms are autosomal recessive and arise due to homozygous pathogenic variants in the genes, which alter collagen transport, folding, post-translational modification, bone mineralization, and cell signaling and bone cell function (Marini et al., 2017). However interconnections between OI phenotype and genotype exist to a certain extent, many carriers of the same mutations might develop different phenotypes and the factors influencing additional phenotype modification remain unidentified (Marini et al., 2007).

Interviews were conducted with 94 Ukrainian OI families in order to gather genealogical information and clinical history and to describe the phenotypes of affected individuals. A *COL1A1/2* mutational analysis of the youngest affected member of each family was performed, to reveal the spectrum of collagen type I pathogenic variants in the Ukrainian OI population. Afterwards, genotype-phenotype correlations in the Ukrainian OI cohort were examined.

This current study presents for the first time the clinical and molecular characteristics of the Ukrainian OI population. Ukrainian OI population enriches pool of known *COL1A1/2* pathogenic variants with 27 novel pathogenic variants. We suppose that this data may advance the understanding of the OI genetic epidemiology; broaden current knowledge about the spectrum of *COL1A1/2* pathogenic variants, patient’s phenotypes, and clinical manifestations; and help to estimate the scope of genotype-phenotype correlations.

## Subjects and Methods

Medical interviews were conducted with 143 individuals affected with OI, from 94 Ukrainian families. Patients were classified according to the updated Sillence OI classification types I–V (Warman et al., 2011). Clinical history collected by reviewing available medical documents and by patients declared medical history. Clinical examination and phenotypes description was done by UT medical team. Phenotypes were described on the basis of observation and available clinical documentation. Mutational analysis of the *COL1A1/2* genes was conducted with Sanger sequencing, in order to reveal the *COL1A1/2* mutational spectrum. OI skeletal and extraskeletal manifestations were compared with pathogenic variant type, to determine genotype-phenotype correlations.

The current study was conducted in accordance with the Helsinki Declaration and received approval from the Sytenko Institute of Spine and Joint Pathology of the Ukrainian Academy of Medical Sciences and the Ethical Review Committee on Human Research of the University of Tartu (permit no. 221/M- 34). Informed written consent from the patients or their legal representatives was obtained prior to inclusion in the study.

### Subjects

Ukrainian OI families from the Ukrainian Association of Crystal People participated in the study. In May 2016 and September 2017, Ukrainian OI patients and their relatives (from all regions of Ukraine) attended an interview and clinical examination with researchers from the University of Tartu, Estonia, in cooperation with Ukrainian medical staff. Patients with other skeletal disorders were excluded from the study during screening (five families).

A total number of 143 unrelated OI patients (66 males and 77 females; aged from 2 months to 65 years) from 94 unrelated families were included in the study. Mutational analysis of the *COL1A1/2* genes was performed on the youngest affected member of every OI kindred included in the study (*n* = 94).

### Clinical Characteristics and Genealogical Description

In order to characterize clinical OI manifestations, patients underwent both clinical and physical examinations. Cases were classified as OI types I–V, according to the observed clinical features, based on severity of the symptoms (Sillence et al., 1979). Patients with mild, non-deforming OI were classified as type I. Patients with moderate variable OI were indicated as type IV. Patients with severe progressive deforming OI were designated as type III. Individuals with signs of calcification of interosseous membranes were enrolled as type V.

Clinical data was registered based on medical documentation. Genealogical data was recorded from the patients’ spoken words. Blood samples were obtained (for DNA analysis) from all available affected family members and their close healthy relatives.

Phenotype description was assessed by clinical observation. Skeletal fractures and deformations (severity, location) and extra-skeletal OI features (sclera color, DI) were noted. All sclera shades on the blue-gray scale were defined as “blue.” Phenotypic data was provided by the medical records, patients, and their relatives, including birth data (weight, height, intrauterine and birth fractures, preterm pregnancy); anthropometric data (weight and height); fracture history (time and location of the first fracture, total number of fractures, number of fractures per year); patient physical mobility; occurrence of hearing loss; and joint laxity. In order to exclude bias, the registration of the phenotype and the OI type classification were performed by a single medical professional. Fracture per year and total fracture values are presented to make data comparable with previous studies. Data regarding additional OI symptoms and features, including pulmonary function, cardiovascular system, BMD was incomplete and incomparable thus excluded from analysis in current study.

Genealogical data included OI history in the family, consanguinity data, and miscarriages. Pedigree trees were constructed for every kindred with the “Kinship2” package of the R statistical program v3.3.2. (R team, Austria).

The Shapiro–Wilk’s test was used to check normality of continuous variables. Normally distributed continuous data was presented as mean and the standard deviation (SD), continuous data lacking normal distribution is presented as median and range. The Student’s *t*-test was used to compare normally distributed continuous data, and Mann–Whitney *U* test was used to compare data without normal distribution.

The categorical data was expressed as percentages. The significance of associations between the genotype and phenotype manifestations was tested with Fisher’s χ^2^-test for categorical variables. *P*-values less than 0.05 were considered to be statistically significant. All statistical analyses were performed using R v3.3.2. software (R Team, Austria) (Chen et al., 2012).

### Mutational Analysis of the *COL1A1/2* Genes

Genomic DNA purification was done with 3 ml of an EDTA-preserved whole blood sample, using the Gentra Puregene Blood Kit (Qiagen, Germany) in accordance with the manufacturer’s protocol and stored at −80°C. gDNA samples were amplified with PCR using a 25 specially designed primer pairs covering the 5’ and 3’ untranslated region and 51 exons of the *COL1A1* gene; 36 primer pairs covering the 5’ UTR and 3’ UTR regions and 52 exons of the *COL1A2* gene. The PCR reaction was performed in a total volume of 20 μl, which included 4 μl of 5× HOT FIREPol^®^ Blend.

Master Mix Ready to Load with 7.5 mM MgCl2 (Solis BioDyne, Estonia), 1 μl each of forward and reverse primer (5 pmol), and 1 μl of gDNA (50 ng). PCR reaction was performed with a Thermal Cycler (Applied Biosystems, USA) PCR machine. The PCR touchdown program has been previously described (Ho Duy et al., 2016; Zhytnik et al., 2017). PCR products were electrophoresed through a 1.5% agarose gel, to control the quality of fragments. The PCR products then purified with exonuclease I and shrimp alkaline phosphatase (Thermo Fisher Scientific, USA). Sanger sequencing reactions were performed on the purified PCR fragments using a BigDye^®^ Terminator v3.1 Cycle Sequencing Kit (Applied Biosystems, USA). Reactions were processed on the ABI3730xl instrument. Applied Biosystems’ Sequence Scanner v1.0 and Mutation Surveyor DNA Variant analysis software v5.0.1. (SoftGenetics, USA) were used to analyze sequence products. Sequence products were further aligned to the GenBank human reference genome sequences of *COL1A1* (gDNA NG_007400.1, complementary (cDNA) NM_000088.3) and *COL1A2* (gDNA NG_007405.1, cDNA NM_000089.3). Sequencing data is available from the authors upon reasonable request. This study focused on the non-synonymous and splice-site variants absent from the publicly available normal datasets (including dbSNP135 and the 1000 Genomes Project) (Consortium T 1000 GP, 2015; Sherry et al., 2001). PolyPhen-2, SIFT, and MutationTaster software tools were used to assess the pathogenic nature and functional impact of discovered variants (Kumar et al., 2009; Adzhubei et al., 2010; Schwarz et al., 2010). Variants not described in osteogenesis imperfecta variant database (http://www.le.ac.uk/ge/collagen/) were classified as novel (Dalgleish, 1997; Dalgleish, 1998; Ho Duy et al., 2016; Zhytnik et al., 2017). Mutational analysis of the patients with OI type V will be reported elsewhere.

All laboratory procedures and data analyses were performed at the University of Tartu, Estonia. The datasets used and analyzed during the current study are available from the corresponding author on reasonable request.

## Results

### Clinical Characteristics of Ukrainian OI Population

**Figure 1:**
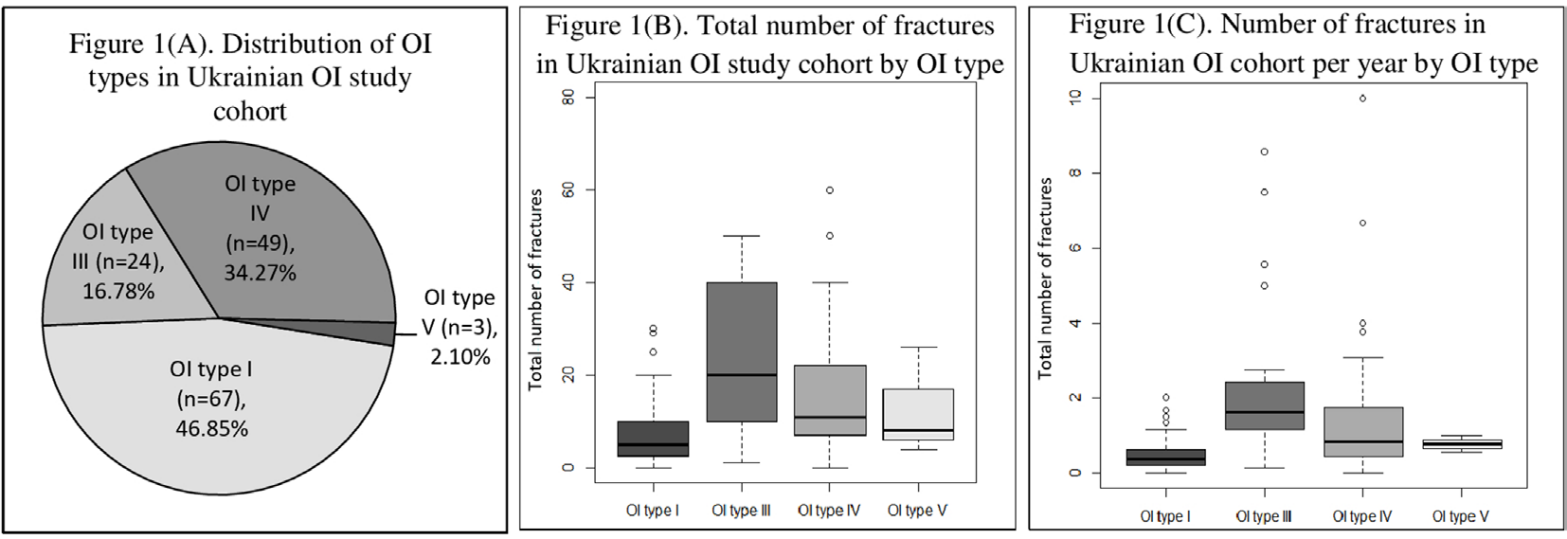
**(A)** Distribution of osteogenesis imperfecta (OI) types in Ukrainian OI study cohort. **(B)** Total number of fractures in Ukrainian OI study cohort by OI type. **(C)** Number of fractures in Ukrainian OI study cohort per year by OI type.

A total number of 143 individuals from 94 unrelated OI families (66 males and 77 females) were included in the study. Within this cohort, there were no cases of consanguineous families. Out of 93 families with known family history, 36 families had a previous history of OI. For one subject, there was no parent information, as he had grown up in an orphanage. Patients were classified in accordance with the new Sillence classification, as follows: type I, *n* = 67 (46.85%); type III, *n* = 24 (16.78%); type IV, *n* = 49 (34.27%); and type V, *n* = 3 (2.10%). In the current study, there were no cases of OI type II (**Figure 1A**).

The age range of the patients was from 2 months to 65 years, with a mean age of 19.5 ± 14.4 years. Slightly more than half of the study subjects (*n* = 77, 54.23%) were in the “child” age group (i.e. between 0 and 17 years). A detailed description of the skeletal and nonskeletal clinical characteristics of the Ukrainian patient cohort is present in **Table 1**.

**Table 1:** Clinical characteristics of the study cohort of Ukrainian osteogenesis imperfecta (OI) patients divided by clinical OI type.

	OI type I	OI type III	OI type IV	OI type V	All OI types
Individuals	67 (46.85%)	24 (16.78%)	49 (34.27%)	3 (2.10%)	143 (100%)
Families with OI history (*n* = 93)	19 (52.78%)	2 (8.70%)	14 (42.42%)	1 (50%)	36 (38.71%)
Gender male/female	35/32	4/20	25/24	2/1	66/77
Age, years (mean ± SD, range) (*n* = 142)	20.3 ± 15.9 (2–65y)	16.3 ± 11.0 (2m–35y)	20.0 ± 13.9 (6m–50y)	17.7 ± 15.2 (4–34y)	19.5 ± 14.4 (2m–65y)
Children (0–17y) 0–5y 6–10y 10–17y	37 (55.22%) 17 (25.37%) 8 (11.94%) 12 (17.91%)	13 (54.17%) 4 (16.67%) 5 (20.83%) 4 (16.67%)	25 (51.02%) 8 (16.33%) 8 (16.33%) 9 (18.37%)	2 (66.67%) 1 (33.33%) 0 (0.00%) 1 (33.33%)	77 (54.23%) 30 (20.98%) 21 (14.69%) 26 (18.18%)
Total fracture number (*n* = 142) (median [range]/mean ± SD)	5.00/(0–30)	20.00/(1–300)	11.00/(0–60)	12.67 ± 11.72	10.00/(0–300)
Fractures per year (*n* = 142) (median [range]/mean ± SD)	0.41 (0–2)	1.63 (0.14–30)	0.83 (0–10)	0.77 ± 0.23	0.61 (0–30)
Time of the first fracture (*n* = 141) No fractures Intrauterine During delivery ≤1y 1–2y 3–6y ≥7y	6 (8.96%) 0 (0.00%) 6 (8.96%) 11 (16.42%) 19 (28.36%) 14 (20.90%) 11 (16.42%)	0 (0.00%) 7 (30.43%) 6 (26.09%) 6 (26.09%) 1 (4.35%) 3 (13.04%) 0 (0.00%)	1 (2.08%) 2 (4.17%) 6 (12.50%) 14 (29.17%) 14 (29.17%) 7 (14.58%) 4 (8.33%)	0 (0.00%) 0 (0.00%) 1 (33.33%) 1 (33.33%) 1 (33.33%) 0 (0.00%) 0 (0.00%)	7 (4.96%) 9 (6.38%) 19 (13.48%) 32 (22.70%) 35 (24.82%) 24 (17.02%) 15 (10.64%)
Most fractured body compartment (*n* = 134) All tubular bones Lower limb Upper limb Phalanx Spine Ribs	9 (14.75%) 25 (40.98%) 20 (32.79%) 6 (9.84%) 1 (1.64%) 0 (0.00%)	3 (13.04%) 18 (78.26%) 2 (8.70%) 0 (0.00%) 0 (0.00%) 0 (0.00%)	11 (23.40%) 27 (57.45%) 7 (14.89%) 0 (0.00%) 0 (0.00%) 2 (4.26%)	0 (0.00%) 2 (66.67%) 1 (33.33%) 0 (0.00%) 0 (0.00%) 0 (0.00%)	23 (17.16%) 72 (53.73%) 30 (22.39%) 6 (4.48%) 1 (0.75%) 2 (1.49%)
Deformations (*n* = 141) Upper limb Lower limb Spine Chest	32 (47.76%) 45 (67.16%) 40 (59.70%) 6 (8.96%)	21 (91.30%) 23 (100.00%) 23 (100.00%) 22 (95.65%)	38 (79.17%) 45 (93.75%) 44 (91.67%) 19 (39.58%)	2 (66.67%) 2 (66.67%) 2 (66.67%) 2 (66.67%)	93 (65.96%) 115 (81.56%) 109 (77.30%) 49 (34.75%)
Mobility (*n* = 140) Immobile Wheelchair Walking with support Walking independently	0 (0.00%) 0 (0.00%) 2 (2.99%) 66 (98.51%)	1 (4.35%) 13 (56.52%) 9 (39.13%) 0 (0.00%)	0 (0.00%) 4 (8.51%) 7 (14.89%) 36 (76.60%)	1 (33.33%) 0 (0.00%) 0 (0.00%) 2 (66.67%)	2 (1.43%) 17 (12.14%) 18 (12.86%) 104(74.29%)
DI (yes) (*n* = 139)	28 (41.79%)	17 (70.83%)	30 (61.22%)	1 (33.33%)	76 (54.68%)
Blue sclera (yes)	57 (85.07%)	20 (86.96%)	44 (89.80%)	3 (100%)	124(87.32%)
Hearing loss (yes)	15 (22.38%)	5 (20.83%)	11 (22.45%)	1 (33.33%)	32 (22.38%)
Time of hearing loss start (*n* = 32) at birth 1–10y 11–20y 21–30y 31–40y	1 (6.67%) 5 (33.33%) 5 (33.33%) 3 (20.00%) 1 (6.67%)	1 (20.00%) 2 (40.00%) 2 (40.00%) 0 (0.00%) 0 (0.00%)	1 (9.09%) 5 (45.45%) 1 (9.09%) 4 (36.36%) 0 (0.00%)	0 (0.00%) 0 (0.00%) 1 (100.00%) 0 (0.00%) 0 (0.00%)	3 (9.38%) 12 (37.50%) 9 (28.13%) 7 (21.88%) 1 (3.13%)

Data are n (%) unless otherwise indicated. SD is standard deviation.

**Figure 2:**
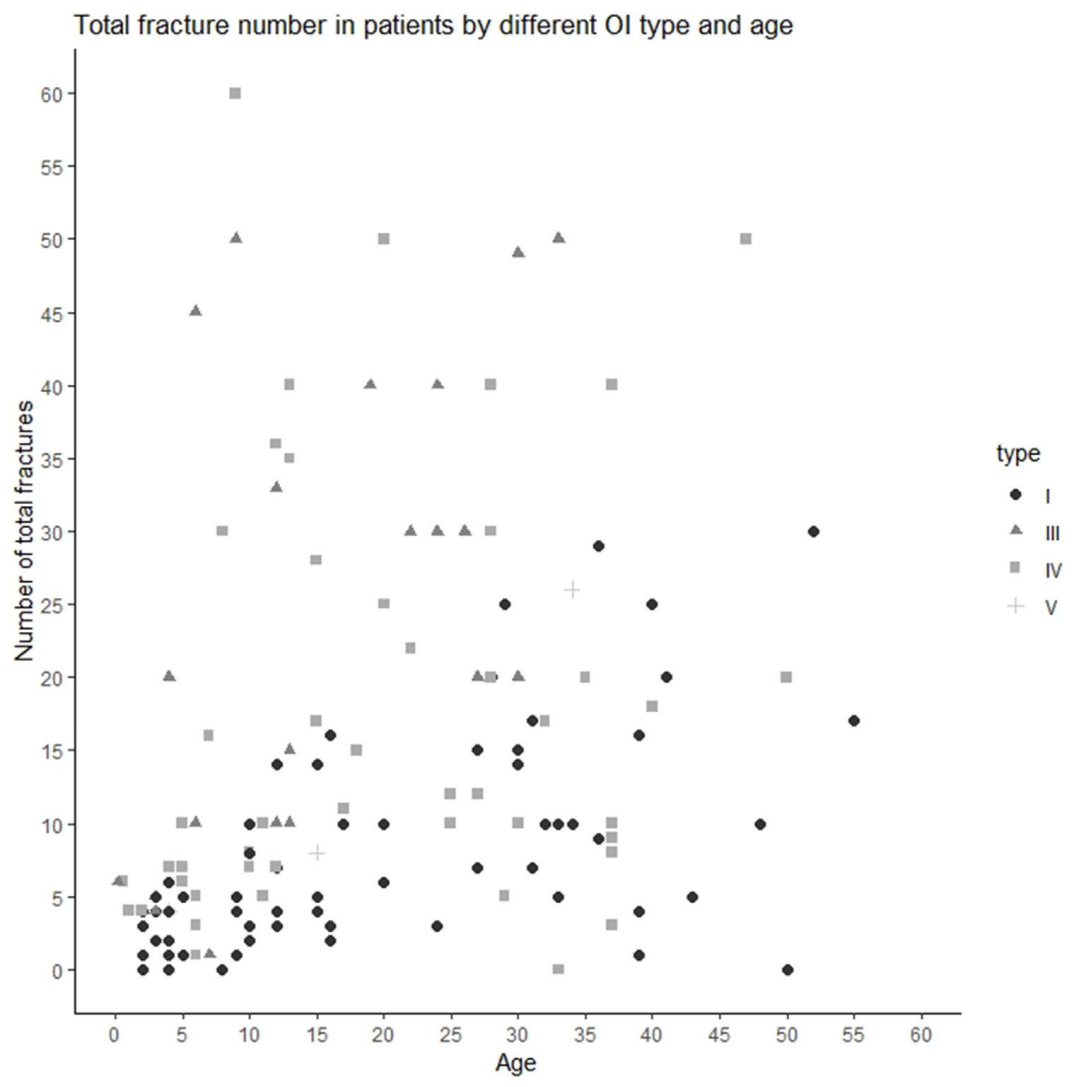
Total number of fractures in Ukrainian OI patient by OI type and age.

The median/(range) of total number of fractures per patient was 10.00 (0–300) (type I 5.00 [0–30], type III 20.00 [1–300], type IV 11.00 [0–60], and type V [median ± SD] 12.67 ± 11.72) (**Figures 1B** and**2**). The number of fractures per year per patient was 1.32 ± 2.88 (type I 0.50 ± 0.43, type III 3.51 ± 6.18, type IV 0.83 ± 1.76, and type V 0.77 ± 0.23) (**Figure 1C**). The highest number of fractures in an individual patient (*n* = 300) occurred in a 35-year old patient with OI type III. Nine patients suffered intrauterine fractures (type III 30.43%, *n* = 7; type IV 4.17%, *n* = 2). 19 patients suffered their first fracture during delivery (type I 8.96%, *n* = 6; type III 26.09%, *n* = 6; type IV 12.50%, *n* = 6; type V 33.33%, *n* = 1). The ages of first fracture for study participants was between 0 and 1 years for 22.70% of patients (*n* = 32); between 1 and 2 years for 24.82% (*n* = 35); between 3 and 6 years for 17.02% (*n* = 24); and at ≥7years for 10.64% (*n* = 15). Seven patients (4.96%) did not experience fractures; of these, one was 50 years old (type I) and one was 33 years old (type IV). However, based on extraskeletal features, skeletal deformations, and positive OI history in the family, both these individuals were diagnosed with OI. The most commonly fractured bones were tubular bones: lower limbs (53.73%, *n* = 72); upper limbs (22.39%, *n* = 30); and both lower and upper limbs (17.16%, *n* = 23).

Deformities were present in the majority of the affected study participants (87.23%, *n* = 123). Type I patients mostly had either no deformities or mild deformities. The majority (81.56%, *n* = 115) of subjects had deformities of the lower limbs of varying severity: severe *n* = 21 (type III, *n* = 16; type IV, *n* = 4; type V, *n* = 1); moderate *n* = 47 (type I, *n* = 12; type III, *n* = 7; type IV, *n* = 27, type V, *n* = 1); and mild (type I, *n* = 33, type IV, *n* = 14). Spine deformations (scoliosis and kyphosis) were present in 77.30% (*n* = 109) of patients. More than half of the patients suffered from deformations of the upper limbs (65.96%, *n* = 93). Approximately one third of patients had chest deformities (34.75%, *n* = 49). Most patients (74.29%, *n* = 104) were able to walk independently; 12.86% (*n* = 18) were able to walk with a support; and 12.14% (*n* = 17) used a wheelchair. Two patients were immobile (type III).

DI was observed in 54.68% (*n* = 76) of the subjects (type I, 41.79%; type III, 70.83%; type IV, 61.22%; type V, 33.33%). Joint laxity was present in 26.62% (*n* = 37) of patients (type I, 24.24%; type III, 27.27%; type IV, 26.53%; type V, 66.67%). Two patients (types I and III) had contractures. The majority of the studied individuals (124 individuals, 87.32%) had blue eye sclera (type I, 85.07%; type 3, 86.96%; type 4, 89.80%; and type V, 100%).

Hearing loss was noted in 22.38% of patients (*n* = 32). Of these, 22.38% were type I (*n* = 15), 20.83% were type III (*n* = 5), 22.45% were type IV (*n* = 11), and 33.33% were type IV (*n* = 1). Congenital hearing loss was diagnosed in three patients (types I, III, and IV). Early hearing loss (at between 1 and 10 years) was noted in 12 patients. Nine patients had lost hearing between the ages of 11–20 years. Eight patients experienced hearing loss from maturity (aged over 20 years).

### Spectrum of *COL1A1/2* Pathogenic Variants in the Ukrainian OI Population

**Figure 3:**
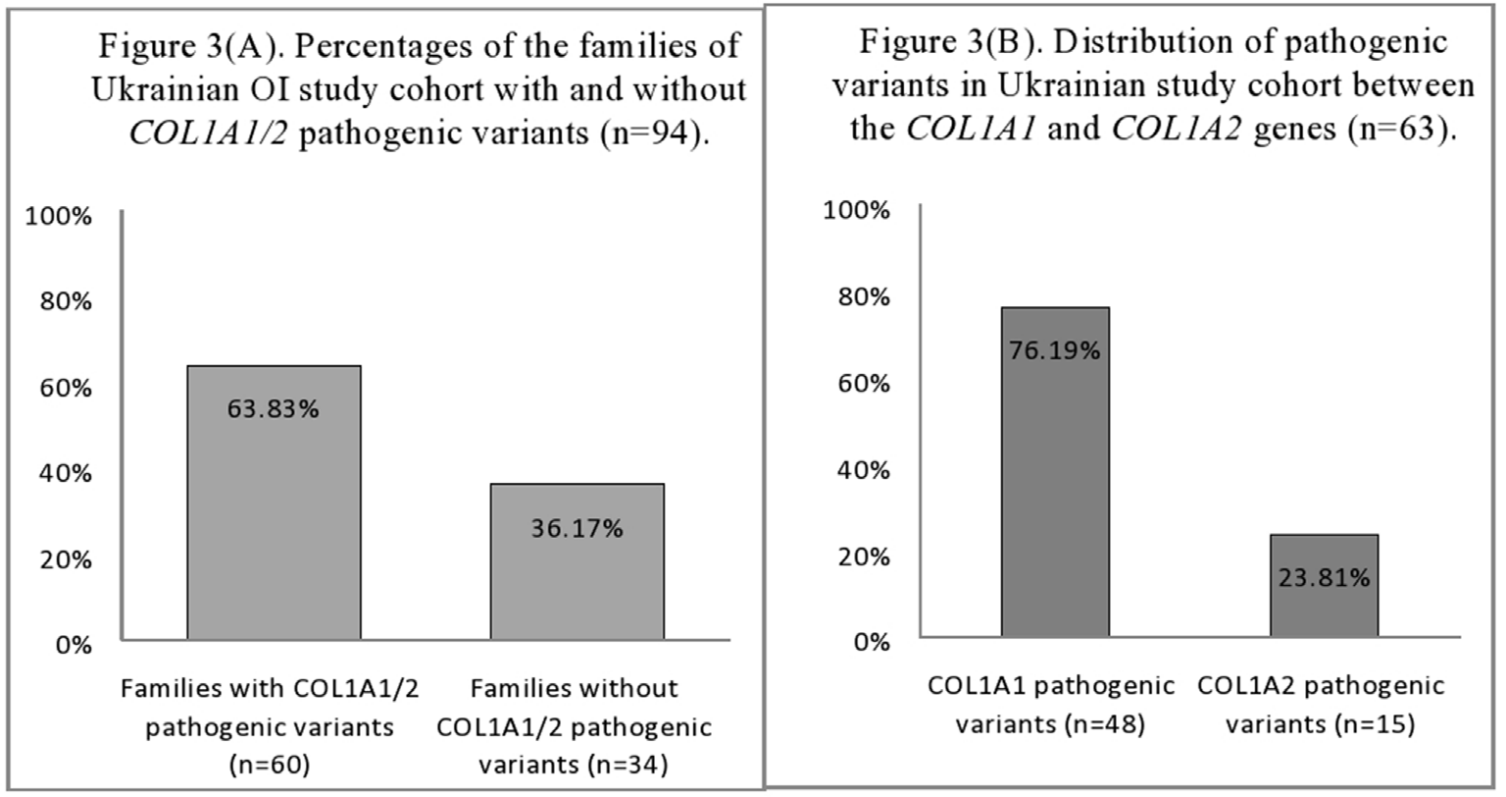
**(A)** Percentages of the families of Ukrainian OI study cohort with and without *COL1A1/2* pathogenic variants (*n* = 94). **(B)** Distribution of pathogenic variants in Ukrainian OI study cohort between the *COL1A1* and *COL1A2* genes (*n* = 63).

The mutational analysis highlighted that 60 (63.83%) of the 94 Ukrainian OI families harbored pathogenic *COL1A1/2* variants (**Figure 3A**). The number of patients harboring *COL1A1/2* pathogenic variants by OI type was as follows: type I, 23 (63.89%); type III, 14 (60.87%); type IV, 23 (69.70%). The number of pathogenic variants was 63, as three patients harbored double pathogenic variants (UA08, UA85 in both *COL1A1/2*; UA55 in *COL1A1*). A list of all observed variants is given in **Table 2**.

**Table 2:** The *COL1A1* (gDNA NG_007400.1, cDNA NM_000088.3) and *COL1A2* (gDNA NG_007405.1, cDNA NM_000089.3) mutational spectrum among studied Ukrainian OI families.

#	Patient ID	Gene	Pathogenic variant	Exon	Pathogenic variant type	Protein alteration	Protein structural domain	OI type
&emsp;1.&emsp;*COL1A1* pathogenic variants								
&emsp;&emsp;a.&emsp;Haploinnsufficiency *COL1A1* pathogenic variants								
1	UA48†	COL1A1	c.1675_delG‡	25	Frameshift	p.Ala559Profs*21	Helical	I
2	UA91	COL1A1	c.1816_del G‡	26	Frameshift	p.Ala606Leufs*160	Helical	I
3	UA95†	COL1A1	c.3489_del G‡	48	Frameshift	p.Pro1165Leufs*74	Helical	IV
4	UA72†	COL1A1	c.3759_delG‡	50	Frameshift	p.Asp1254Thrfs*77	C-terminal propeptide	I
5	UA15†	COL1A1	c.459_delT	5	Frameshift	p.Gly154Alafs*111	N-terminal propeptide	I
6	UA14†	COL1A1	c.579_delT	7	Frameshift	p.Gly194Valfs*71	Chain	I
7	UA61	COL1A1	c.2393_dupC‡	35	Frameshift	p.Gly800Argfs*5	Helical	I
8	UA67	COL1A1	c.2523_delT	37	Frameshift	p.Gly842Alafs*266	Helical	I
9	UA31	COL1A1	c.2821_delG‡	40	Frameshift	p.Gly941Valfs*167	Helical	IV
10	UA44†	COL1A1	c.1789G > T	26	Nonsense	p.Glu597*	Helical	I
11	UA50	COL1A1	c.3076C > T	43	Nonsense	p.Arg1026*	Helical	I
12	UA19	COL1A1	c.658C > T	9	Nonsense	p.Arg220*	Helical	I
13	UA45†	COL1A1	c.1081C > T	17	Nonsense	p.Arg361*	Helical	IV
14	UA64	COL1A1	c.1243C > T	19	Nonsense	p.Arg415*	Helical	I
15	UA17†	COL1A1	c.1426G > T‡	21	Nonsense	p.Arg476*	Helical	I
16	UA93	COL1A1	c.2179C > T‡	32	Nonsense	p.Gln727*	Helical	IV
17	UA73†	COL1A1	c.3807G > A	49	Nonsense	p.Trp1269*	C-terminal propeptide	I
18	UA22†	COL1A1	c.495T > A‡	6	Nonsense	p.Tyr165*	Helical	I
19	UA25	COL1A1	c.904-9G > A	13i	Splice site	–	–	I
20	UA38	COL1A1	c.2613+6T > C	38i	Splice site	–	–	IV
21	UA40†	COL1A1	c.1614+1G > A	23i	Splice site	–	–	IV
22	UA41†	COL1A1	c.3815−1G > A‡	48i	Splice site	–	–	IV
23	UA43†	COL1A1	c.858+1G > A	12i	Splice site	–	–	IV
24	UA56†	COL1A1	c.858+1G > A	12i	Splice site	–	–	IV
25	UA80	COL1A1	c.804+1G > A	11i	Splice site	–	–	IV
&emsp;&emsp;b.&emsp;Structural *COL1A1* pathogenic variants								
26	UA101	COL1A1	c.3226G > A	45	Missense	p.Gly1076Ser	Helical	IV
27	UA23	COL1A1	c.769G > A	11	Missense	p.Gly257Arg	Helical	I
28	UA27†	COL1A1	c.2101G > T	31	Missense	p.Gly701Cys	Helical	III
29	UA62	COL1A1	c.1057G > T	17	Missense	p.Gly353Cys	Helical	IV
30	UA39	COL1A1	c.3652G > A	49	Missense	p.Ala1218Thr	Helical	IV
31	UA57†	COL1A1	c.757C > T	11	Nonsense	p.Arg253*	Helical	IV
32	UA21†	COL1A1	c.3655G > T‡	49	Missense	p.Asp1219Tyr	C-terminal propeptide	IV
33	UA71†	COL1A1	c.4356G > C‡	52	Missense	p.Gln1452His	C-terminal propeptide	III
34	UA89†	COL1A1	c.653G > A	9	Missense	p.Gly218Asp	Helical	I
35	UA53†	COL1A1	c.734G > A‡	10	Missense	p.Gly245Glu	Helical	I
36	UA33†	COL1A1	c.742G > A	10	Missense	p.Gly284Arg	Helical	III
37	UA30†	COL1A1	c.1319G > C‡	20	Missense	p.Gly440Ala	Helical	III
38	UA76†	COL1A1	c.1192G > A‡	18	Missense	p.Gly398Ser	Helical	III
39	UA94†	COL1A1	c.1588G > A	23	Missense	p.Gly530Ser	Helical	III
40	UA10	COL1A1	c.2362G > A	35	Missense	p.Gly788Ser	Helical	IV
41	UA78	COL1A1	c.2434G > A‡	36	Missense	p.Gly812Ser	Helical	IV
42	UA05†	COL1A1	c.2461G > A	37	Missense	p.Gly821Ser	Helical	III
43	UA96	COL1A1	c.2560G > A	38	Missense	p.Gly854Ser	Helical	I
44	UA32	COL1A1	c.1A > C‡	1	Missense	p.Met1Leu	Signal peptide	I
&emsp;2.&emsp;*COL1A2* pathogenic variants								
&emsp;&emsp;a.&emsp;Haploinsufficiency *COL1A2* pathogenic variants								
45	UA82	COL1A2	2093_2110_dup‡	35	Frameshift	p.Leu699_Leu704dup	Helical	III
46	UA92†	COL1A2	c.2026-1_2042_dup‡	33i-34	Splice site	–	–	III
&emsp;&emsp;b.&emsp;Structural *COL1A2* pathogenic variants								
47	UA86†	COL1A2	c.2045G > T‡	34	Missense	p.Gly682Val	Helical	III
48	UA37	COL1A2	c.2233G > C	37	Missense	p.Gly745Arg	Helical	III
49	UA83†	COL1A2	c.2027G > A	34	Missense	p.Gly676Asp	Helical	IV
50	UA51†	COL1A2	c.3034G > A	46	Missense	p.Gly1012Ser	Helical	III
51	UA54†	COL1A2	c.982G > A	19	Missense	p.Gly328Ser	Helical	III
52	UA65	COL1A2	c.1009G > A	19	Missense	p.Gly337Ser	Helical	IV
53	UA102†	COL1A2	c.2224G > A‡	37	Missense	p.Gly742Arg	Helical	I
54	UA74	COL1A2	c.2288G > T	37	Missense	p.Gly763Val	Helical	III
55	UA47	COL1A2	c.2314G > A	38	Missense	p.Gly772Ser	Helical	IV
56	UA69†	COL1A2	c.2324G > A	38	Missense	p.Gly775Glu	Helical	IV
57	UA90†	COL1A2	c.1220T > C‡	22	Missense	p.Leu407Pro	Helical	I
&emsp;3.&emsp;Double pathogenic variants in the *COL1A1* and *COL1A2*								
58	UA55†	COL1A1	c.2195_delA‡	32	Frameshift	p.Glu732Aspfs*34	Helical	IV
		COL1A1	c.2548_2549_dupC‡	36	Frameshift	p.Gly851Luefs*258	Helical	
59	UA85	COL1A1	c.370-1G > A	4i	Splice site	-	-	I
		COL1A2	c.2642A > C‡	41	Missense	p.Glu881Ala	Helical	
60	UA08†	COL1A1	c.334-1G > A‡	3i	Splice site	-	-	IV
		COL1A2	c.2642A > C‡	41	Missense	p.Glu881Ala	Helical	

Patients with sporadic pathogenic variants are marked with an obelisk (†). Novel pathogenic variants unreported in the collagen type I variant database (http://www.le.ac.uk/ge/collagen/) are marked with a diesis (‡).

The number of *COL1A1* pathogenic variants was 48/63 (76.19%), whereas in the *COL1A2* gene, 15/63 (23.81%) variants were observed (**Figure 3B**). All pathogenic variants were in a heterozygous state, underlying the dominant inheritance pattern of the disorder in this cohort. Novel pathogenic variants were represented by 27 (42.85%) variants (20 in *COL1A1*; 7 in *COL1A2*) absent from the *osteogenesis imperfecta* variant database (**Table 2**).

**Figure 4:**
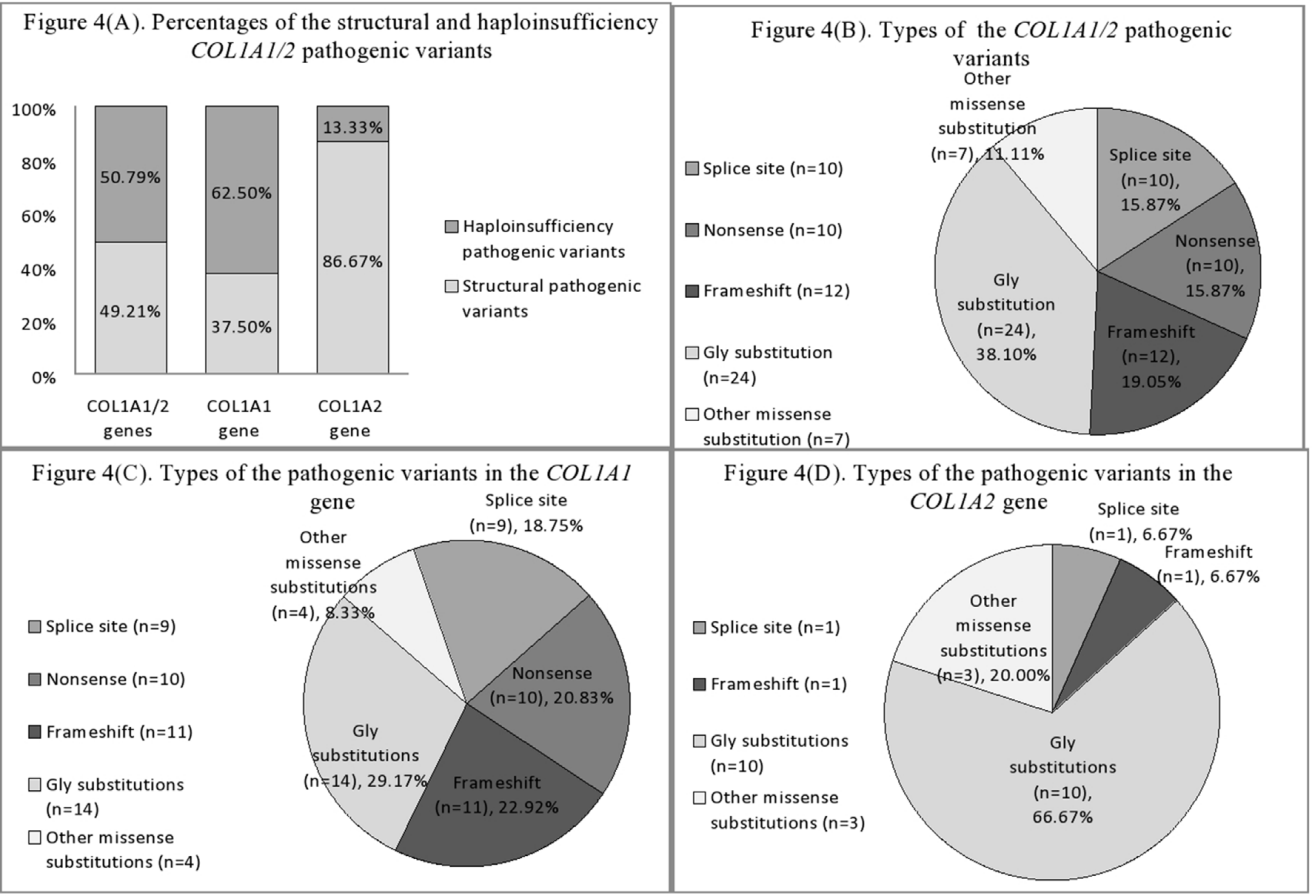
**(A)** Percentages of the structural and haploinsufficiency *COL1A1/2* pathogenic variants in Ukrainian OI study cohort. **(B)** Mutational spectrum of the *COL1A1/2* genes in Ukrainian OI study cohort. **(C)** Types of pathogenic variants in the *COL1A1* gene in Ukrainian OI study cohort. **(D)** Types of pathogenic variants in the *COL1A2* gene in Ukrainian OI study cohort.

Structural variants comprised slightly more than half of revealed pathogenic variants (31/63, or 49.21%), whereas haploinsufficiency variants were observed in 32/63 (50.79%) of cases (**Figure 4A**).

Of the 31 structural pathogenic variants, 18 (58.06%) were situated in the *COL1A1* gene and 13 (41.94%) in the *COL1A2* gene. Glycine substitution was present in 24 (77.42%) of the missense variants, of which 14 (58.33%) and 10 (41.67%) were in the *COL1A1* and *COL1A2* genes, respectively. Glycine substitution with serine was present in 11 (45.83%) Gly missense variants, seven in the *COL1A1*, and four in the *COL1A2* genes (**Figures 4B–D**). Two individuals harbored missense non-Gly substitution pathogenic variants in the C-terminal propeptide of the collagen α1 chain: UA21 c.3655G > GT (p.[Asp1219Tyr]) and UA71 c.4356G > GC (p.[Gln1452His]).

Thirty (93.75%) of the haploinsufficiency variants arose in the *COL1A1* gene and 2 (6.25%) in the *COL1A2*. Frameshift pathogenic variants were represented by 12 (37.50%) variants, 11 (91.67%) in the *COL1A1* gene, and one (8.33%) in the *COL1A2*. Furthermore, 11 (34.38%) splice site variants were discovered (nine of *COL1A1*; one of *COL1A2*), as well as 10 (31.25%) nonsense variants, all of which altered the *COL1A1* gene (**Figures 4B–D**).

### Correlation Between Clinical Characteristics and Genotypes of Ukrainian OI Patients

The results of the analyses show a clear correlation between OI type and collagen defect. Haploinsufficiency collagen pathogenic variants correlate with milder OI types (*p* = 0.007). Interestingly, haploinsufficiency pathogenic variants in the *COL1A2* gene both caused severe OI, whereas the *COL1A1* variants caused moderate and mild OI (*p* = 0.003). However, there was no difference in OI types between structural *COL1A1* and *COL1A2* pathogenic variants (*p* = 0.895) (**Table 3**).

**Table 3:** Relationships between clinical characteristics and collagen I pathogenic variant types for cohort of Ukrainian OI patients.

	Structural OI type I/haploin­sufficiency OI type I	All structural/all haploin­sufficiency	Haploin­sufficiency *COL1A1*/haploin­sufficiency *COL1A2*	Structural *COL1A1*/structural *COL1A2*	Gly *COL1A1*/Gly *COL1A2*	Gly > Ser *COL1A1*/Gly > Ser *COL1A2*	Collagen OI/non-collagen OI
**All OI patients** *Type I* *Type III* *Type IV*	7/16 – – –	29/31 7/16 12/2 10/13	27/2 15/0 0/2 12/0	18/11 5/2 7/5 6/4	20/10 8/1 6/5 6/4	7/4 1/0 3/2 3/2	60/34 23/13 14/9 23/10
*p*-value	–	0.003*	0.004*	0.895	0.271	1	0.317
**Blue sclera**	5/15	24/30	26/2	15/9	16/8	4/3	54/23
*p*-value	0.481	0.329	1	1	1	1	0.011*
**Mobility** *Wheelchair* *Walking with support* *Walking independently* *Immobile*	– 1/1 5/15 –	8/2 7/5 12/23 –	1/1 5/0 21/0 –	2/6 6/1 9/3 –	1/6 7/1 11/2 –	1/1 3/1 2/2 –	10/5 12/4 35/22 0/1
*p*-value	0.481	0.023*	0.169	0.038*	0.002*	1	0.465
**Deformation severity ** (*p*-values) *Upper limb* *Lower limb* *Spine* *Chest*	0.065 0.022* 0.029* 1	0.001* 5.803e-05* 2.37e-05* 2.0e-04*	0.734 0.048* 0.285 0.598	0.162 0.765 0.224 0.190	0.051 0.138 0.010* 0.004*	0.714 0.2 0.543 0.2	0.558 0.473 0.441 0.125
**Total fracture number** (median [range]/mean ± SD)	6.00/4.00 (4–14)/(0–25)	28.00/6.00 (4–300)/(0–40)	6.00/13.00 (0–40)/(6–20)	10.00/30.00 (4–300)/(7–50)	7.00/30.00/ (2–49)/(7–50)	8.00/12.50 (4–49)/(7–30)	10/6.5 (0–300)/(0–50)
*p*-value	0.1184	0.001*	0.4899	0.3131	0.0247*	0.5918	0.046*
**Fracture number per year** (median [range]/ mean ± SD)	1.00/0.73 (0.4–1.17) (0–2)	1.52/0.83 (0.4–8.57)/ (0–30)	0.8/15.37 (0–2.69)/(0.74–30)	1.63/1.39 (0.4–8.57)/ (0.7–3.08)	1.33/1.52 (0.25–7.5)/ (0.77–3.08)	1.48/0.69 (1–3.75)/(0.78–1.75)	1.18/0.54 (0–30)/(0–5.56)
*p*-value	0.483	0.001*	0.282	0.651	0.538	0.352	0.004*

Statistically significant p-values are marked with *. Data are n, unless otherwise indicated. SD is standard deviation.

Type of pathogenic variant appeared to be crucially important for total fracture number, as well as for number of fractures per year. Patients with structural OI pathogenic variants had more fractures than did patients with haploinsufficiency defect. For those patients with structural defect (Gly substitutions), patients with *COL1A1* pathogenic variants had more total fractures than did *COL1A2* patients (*p* = 0.0247) (**Table 3**).

There were clear correlations between skeletal deformations and collagen defect. Patients with a structural collagen defect suffered from more severe skeletal deformations than did patients with a haploinsufficiency OI defect (*p* = 0.001; *p* = 5.80e-05; *p* = 2.37e-05; *p* = 2.0e-04) (**Table 3**). Interestingly, patients with structural OI type I revealed more severe deformations in the lower limbs and spine, compared to those with haploinsufficiency OI type I (*p* = 0.022, *p* = 0.029). Gly substitutions in *COL1A2* genes led to more severe cases of chest and spine deformations, compared to Gly substitutions in the *COL1A1* gene (*p* = 0.010, *p* = 0.004). Patients with structural pathogenic variants were less mobile than patients with haploinsufficiency pathogenic variants (*p* = 0.023). Also those with structural *COL1A2* pathogenic variants were less mobile than patients with structural *COL1A1* pathogenic variants (*p* = 0.038).

There was no correlation between pathogenic variant type and presence of DI or hearing loss in the cohort of Ukrainian patients. However, the correlation of blue sclera with the presence of collagen I pathogenic variants (*p* = 0.011) was notable, as the majority of non-collagenous OI cases had white eye sclera.

## Discussion

### Phenotype Characteristics of Ukrainian OI Cohort

In current study, the clinical and molecular characteristics of individuals from 94 osteogenesis imperfecta families from Ukraine were presented. Of the 143 subjects, 46.85% were diagnosed with OI type I. 16.78% of the patients had OI type III and 34.27% were described as having type IV. Three patients were classified as OI type V due to enlargement of the hyperplasic callus and were guided toward further analysis of the *IFITM5* gene, which will be presented elsewhere (**Table 1**, and **Figure 1A**). Previous studies showed that, amongst Polish OI patients (*n* = 123), patients with OI types I, III, and IV comprised 44%, 33%, and 21% of the cohort, respectively (Rusinska et al., 2017). In a Russian pediatric study (including data from 31 regions of the Russian Federation), patients (*n* = 117) were classified as follows: OI type I, 59%; type III, 27%; and type IV, 14% (Yakhyayeva et al., 2016).

Interestingly, the current study revealed a low percentage of patients with OI type III. However, this may be connected with the difficulty involved for patients with severe forms of OI forms to travel to the study venue. Nevertheless, OI patients with milder OI types are usually rarer in population cross-sectional studies compared with nationwide register-based studies, due to poor diagnostics and less patient interest. The current study illustrates the effective work of the Ukrainian Association of Crystal People, which enabled the involvement in research of those individuals with milder OI. In studies of other populations, the distribution by types (I/III/IV) were as follows: Vietnamese 31.5%/31.5%/37%, Taiwanese 58%/7%/35%, Chinese 28%/38%/34%, Israeli 61%/21%/14%, Swedish 68%/13%/19%, Norwegian 77%/9%/11%, and Finnish 72%/4%/20% (Hartikka et al., 2004; Lin et al., 2015; Lindahl et al., 2015; Binh et al., 2017). There may be various reasons for the observed contrasts in the proportion of OI types across different populations, such as sample sizes, diagnostics, and classification. Additionally, methods of patient recruitment might affect distribution of OI types in our cohort, as patients were enrolled *via* OI patients’ organization and thus OI individuals with more severe OI forms might be overrepresented in contrast to individuals with mild OI forms. Being a spectrum of disorders, OI type classification remains subjective, as some of the individuals might develop border phenotypes and thus might be classified by different health professionals differently. Current circumstances might add bias and contrast to results of different studies. However, the influence of genetic factors cannot be excluded.

In the current study, the number of fractures per year was strongly aligned with those of a previous Swedish OI study (type I 0.57 ± 0.68; type III 3.83 ± 9.32; and type IV1.33 ± 1.38) (Lindahl et al., 2015). In contrast to Swedish register-based study, clinical features in our cohort were partially self-reported and it might bias the data. However, similar to previous studies, more deformed and fractured body compartments occurred in the lower limbs, which may be due to the greater loading of the lower limbs (Binh et al., 2017). We discovered two adult patients without history of fractures, but with other OI symptoms and OI history in the family. Both cases lacked pathogenic variants in *COL1A1/2* genes. Therefore, it would be particularly interesting to identify causative pathogenic variants in these cases and further investigate reasons of fracture absence.

The proportion of patients suffering from DI (55%) was higher than in Swedish (25%) and Brazilian (27%) OI cohorts, but similar to the proportions in Taiwanese (44%) and Vietnamese (61%) OI populations (Lin et al., 2015;Lindahl et al., 2015;Binh et al., 2017; Brizola et al., 2017). The percentage of patients with blue sclera (87%) aligns with the proportions observed in previous studies (e.g. 82%, 93%, 90%, and 80% of patients in Swedish, Brazilian, Taiwanese, and Vietnamese cohorts, respectively) (Lin et al., 2015; Lindahl et al., 2015; Binh et al., 2017; Brizola et al., 2017).

### Genotype Characteristics of Ukrainian OI Cohort

According to previous studies, collagen I pathogenic variants account for between 60 and 90% of OI cases (Dalgleish, 1998). In a recent major genetic study of 598 OI individuals from 487 families of Caucasian, Hispanic, Arab, and Asian origin, the proportion of collagen pathogenic variants was 86% (Bardai et al., 2016). However, in population-based OI studies, *COL1A1/2* percentages vary. In this current study, the proportion of *COL1A1/2* pathogenic variants in Ukrainian OI patients comprised 63.83%, which appears to be lower than in Northern Europe [e.g. in Estonian and Swedish (87%) and Finnish (91%) populations] (Hartikka et al., 2004; Lindahl et al., 2015; Zhytnik et al., 2017). At the same time, these results show that Ukrainian OI patients harbor a higher number of *COL1A1/2* pathogenic variants than do patients from Russia and Asian populations. Amongst patients from Russia (Yakutia and Bashkortostan regions), the percentage of collagen I pathogenic variants was 41% (Khusainova et al., 2012). Asian populations from Vietnam, Taiwan, and Korea were characterized by *COL1A1/2* pathogenic variants of 59%, 51%, and 52%, respectively (Lee et al., 2006; Lin et al., 2015; Ho Duy et al., 2016).

We have previously analyzed Estonian OI patients, using same analysis methods and laboratory techniques (Zhytnik et al., 2017). Regarding the fact, that proportion of the *COL1A1/2* mutations in the Estonian OI cohort composed ∼90%, we suppose that analysis methods and techniques could not influence proportion of the collagen I mutations in the Ukrainian OI cohort.

Panel sequencing of the autosomal recessive OI genes in Ukrainian OI patients without collagen I mutations is ongoing and will be reported elsewhere. Patients with contractures will be scanned for presence of variants in the *PLOD2* and *FKBP10* genes to specify presence of a Bruck syndrome diagnosis.

The current analysis also illustrated that the proportion of pathogenic variants of the *COL1A2* gene was less than that of the *COL1A1* (23.81% and 76.19%, respectively). Similar results were observed in Estonian, Swedish, Finnish, and Taiwanese populations (Hartikka et al., 2004; Lin et al., 2015; Lindahl et al., 2015; Zhytnik et al., 2017). However, in a Russian study of 83 OI patients of Turkic and Slavic origin, and in a study of 11 Egyptian patients, pathogenic *COL1A2* variants were not observed (Khusainova et al., 2012; Aglan et al., 2015).

Another interesting result of the current study was that the amounts of structural and haploinsufficiency variants were almost equal (49.21% and 50.79%, respectively). This was similar to results reported for a Swedish OI cohort (Lindahl et al., 2015). In contrast, in Estonian and Finnish cohorts, the proportions of structural OI pathogenic variants were significantly lower (31% and 30%, respectively), whereas in Taiwanese and Vietnamese cohorts, the proportion was higher (78%) (Lin et al., 2015; Ho Duy et al., 2016; Zhytnik et al., 2017).

The reasons for differences in genotypes may be similar to those for differences in OI type distributions: i.e. sample sizes, methods of patient recruitment, and potential variations in OI genetic epidemiology between different populations.

As expected, the majority of haploinsufficiency variants caused OI types I and IV. Only two individuals with haploinsufficiency variants had OI type III (**Table 2**). Investigation of genotypes in patients with OI type III and *COL1A2* haploinsufficiency variants for presence of mutations in other OI genes is essential and current patients are included into panel sequencing cohort. The majority of patients with structural pathogenic variants were represented by individuals with OI types IV and III. However, seven individuals had OI type I, of which five harbored glycine substitutions. Patients with double pathogenic variants had moderate and mild phenotypes IV and I. Patient UA08 had blue sclera, hearing loss (started at the age of 10). Patients UA55 and UA85 had blue sclera, DI, but no hearing loss. It is known that phenotype severity depends not only on the type of the pathogenic variant, but also on helical location and substituted residue, which contribute to the development of the phenotype (Marini et al., 2007; Rauch et al., 2010). It could be proposed, that patient UA55 who had two frameshift variants in the *COL1A1* gene might have had a haploinsufficiency of the collagen α1 chain and suffer from quantitative collagen defect.

Unexpectedly mild phenotypes were harbored by patients UA89, UA53, UA96, UA23, UA102. All of these patients suffered OI I, however had Gly structural substitutions, which generally are associated with severe OI. Interestingly, *COL1A1* c.769G > A (p.[Gly257Arg] variant (UA23) was reported for 37 times, causing all range of classical non-lethal OI types I, III, IV. Number of observations might have underlined phenotype diversity evidence, stating broader spectrum of affection compared to variants with lack of data. Another *COL1A1* variant c.653G > A (p.[Gly218Asp]) (UA89) was also described in a patient with OI IV. Whereas variant c.2560G > A was previously found in a patients with OI I/IV (Marini et al., 2007). Both patients had apparently mild types and differences in phenotype presentation might be connected to classification bias of border OI forms (i.e. type I/IV). Moreover, phenotypes of OI patients might be affected by the treatment, and real effect of the mutation might remain unresolved.

The above discussion indicates that, although the general effect of collagen I pathogenic variants on phenotype is understood, current knowledge does not reflect all of the nuances of genotype-phenotype correlations. OI is known to show incomplete penetrance, thus the clinical presentation can vary to a great degree even between same family members (Van Dijk and Sillence, 2014). Recent studies show over hundred loci in the human genome influencing bone mineral density (Estrada et al., 2012; Rivadeneira and Mäkitie, 2016). In addition bone morphology and quality, bone strength, and toughness, also influence fracture risk (Chesnut and Rosen, 2001; Rivadeneira and Mäkitie, 2016). Bone is influenced by metabolic (glucose, lipids, calcium, hormones), biomechanical (muscle strain, body weight, and composition), material (collagen, mineralization), cellular (osteoclast, osteoblast, osteocyte activity, and differentiating), growth, and remodeling factors (Rivadeneira and Mäkitie, 2016). All these factors can potentially influence the phenotypic expression of collagen gene mutations by yet undescribed molecular pathways. Further investigations of the clinical manifestations and molecular characteristics of patients may contribute to the understanding of OI phenotypes’ diversity.

In current study we present 27 novel OI pathogenic variants, harbored by Ukrainian OI patients. Some novel pathogenic variants alter previously-reported positions with new substitutions; this could be of particular interest to the exploration of the OI phenotypical spectrum and its interconnections with genotypes. Moreover, current pathogenic variants enrich database of OI pathogenic variants and have practical use for OI genetic diagnostics. The percentage of novel pathogenic variants in Ukrainian OI cohort was 42.85%. Despite the numerous reported variants, there are still many novel collagen type I pathogenic variants, each of which improves the understanding of OI genotype-phenotype correlations.

As it is known, splice site mutations might result in exon skipping, intronic inclusion, or activation of cryptic splice sites (Marini et al., 2007). Changes in mRNA and protein depend on whether these alterations are in frame or produce translational frameshifts. Pathogenic mechanism of the identified novel OI haploinsufficiency variants is yet to be investigated with further functional studies. In general, haploinsufficiency mutations result in a severely truncated mRNA molecules, which cause activation of pretermination stop codon, followed by mediated mRNA decay (Chang et al., 2007; Fang et al., 2013; Symoens et al., 2014).

Structural pathogenic variants cause synthesis of an abnormal protein. Defective protein is secreted into the extracellular matrix and interferes with fibrillogenesis, collagen-matrix, bone cells, and hydroxyapatite. The structural pathogenic variants affect extracellular matrix more severely than haploinsufficiency variants (Bodian et al., 2008; Marini et al., 2017). Although exact mechanism of the structural pathogenic mutations has to be further elucidated with functional studies, according to *in silico* analysis and description of functional domains in the collagen type I protein, following consequences of the novel structural variants can be predicted: *COL1A1*, c.3655G > T, p.(Asp1219Tyr), UA21, OI IV

Substitution in identical position was described previously by Lindahl *et al*. c.3655G > A (p.[Asp1219Asn]) (Lindahl et al., 2011). However, in contrast to Ukrainian patient, Swedish patient had mild OI phenotype. It can be partly explained by the hydrophobic side chain of the Tyr, which compared to uncharged side chain of Asn, has larger effect on procollagen properties.

Both variants alter C-cleavage site, what causes severe processing defect, alteration of the C-propeptide cleavage, and increased mineralization (Lindahl et al., 2011; Marini et al., 2017). However, we do not have bone mineral density data of the UA21 patient to confirm high bone mass phenotype.

*COL1A1*, c.4356G > C, p.(Gln1452His), UA71, OI III

Current structural variant also alters C-cleavage site of the procollagen, what is critical for mineralization. In addition, according to *in silico* analysis, current variant causes gain of donor splice site and might result in a cryptic splice site. Current mutation might be particularly interesting for further functional investigation, as patient suffered more than 300 fractures during lifetime and developed hearing loss at the age of 2.

*COL1A1*, c.734G > A, p.(Gly245Glu), UA53, OI I

According to collagen type I functional domains, current variant might affect keratan sulfate proteoglycans binding region, α2β1 integrin, and interleukin 2 (IL2) binding sites (Sweeney et al., 2008). In this way, variant might alter protein function.

*COL1A1*, c.1319G > C, p.(Gly440Ala), UA30, OI III

Variant affects keratane sulfate proteoglycans binding domain and probably creates a new donor splice site (Sweeney et al., 2008).

*COL1A1*, c.1192G > A, p.(Gly398Ser), UA76, OI III

Variant is located in the proposed site of discoidin domain receptor 2 (DDR2) binding site, von Willebrand Factor binding site, and dermatan/chondroitin sulfate proteoglycans/decorin binding region (Sweeney et al., 2008). Marini et al. has described patient with OI III/IV harboring variant c.1192G > T (p.[Gly398Cys]) (Marini et al., 2007). More severe phenotypes of UA76 patient might be caused by more distant properties of Ser compared to Cys in relation to Gly and have greater effect on protein function.

*COL1A1*, c.2434G > A, p.(Gly812Ser), UA78, OI IV

Current variant is located in the major ligand binding region (MLBR) 2, proposed region for lethal OI mutations (Marini et al., 2007). Variant overlaps with α2β1 integrin binding site and glycation region (Sweeney et al., 2008).

*COL1A1*, c.1A > C, p.(Met1Leu), UA32, OI I

The variant alters initiating methionine, causing activation of potential downstream translation initiation site with new reading frame. Substitutions of the Met in the *COL1A1* were previously described and resulted in OII.

*COL1A2*, c.2045G > T, p.(Gly682Val), UA86, OI III

Variant is located in the MLBR2 and interrupts cartilage oligomeric matrix protein and phosphophoryn, secreted protein, acidic, and rich in cysteine (SPARC) binding sites, keratan sulfate proteoglycans binding region (Sweeney et al., 2008).

*COL1A2*, c.2224G > A, p.(Gly742Arg), UA102, OI I

Variant is located in the MLBR2, overlaps with SPARC binding site, cell interaction domain, cartilage oligomeric matrix protein, phosphoryn binding sites (Sweeney et al., 2008).

*COL1A2*, c.1220T > C, p.(Leu407Pro), UA90, OI I

Variant is located in the dermatan/chondroitin sulfate proteoglycans/decorin binding region.

*COL1A2*, c.2642A > C, p.(Glu881Ala), UA85, OI IV, and UA08, OI I

Variant is located at the delineate clusters of lethal OI mutations on the α2(I) chain. Current variant might alter proposed site of dermatan/chondroitin sulfate proteoglycans/decorin, IL2, amyloid precursor protein binding regions (Sweeney et al., 2008). Both variants are harbored by individuals with additional *COL1A1* variants.

Genotypes were assessed with Sanger sequencing, which is considered to be a powerful and accurate method of mutational analysis. Special design of primers allowed the capture of all inter-exon junction regions and 5’UTR, 3’UTR regions. However, potential limitations include the inability of Sanger sequencing to detect whole gene or exon deletions and duplications. The number of *COL1A1/2* pathogenic variants may therefore be underestimated. Differences in sample sizes may also contribute to the variation of results between studies.

### Genotype-Phenotype Analysis

OI genotype-phenotype correlations have been a subject of interest for many decades. Although the association of pathogenic variant with OI phenotype remains elusive, previous studies showed *COL1A1* pathogenic variants to be associated with more severe phenotypes than were *COL1A2* (Marini et al., 2007; Mrosk et al., 2018). In the current study, a higher fracture number was observed in structural *COL1A1* cases; among *COL1A2* cases there were more cases with severe deformations of the spine and chest.

These results show the presence of a strong correlation between collagen type I defect and severity of OI, which aligns well with previous studies. Haploinsufficiency collagen I defects cause milder, less fragile, and less deformed OI types (Marini et al., 2007; Amor et al., 2011; Lindahl et al., 2015). Although in a Swedish OI cohort, the difference between type I patients with structural and haploinsufficiency collagen defects were insignificant, in the current study, structural type I patients had more deformed lower limbs and more fractures. This underlies the vital importance of a normal collagen helical structure (rather than amount).

In contrast to previous studies, the current study did not find an association between DI and pathogenic variant type. This apparent lack of correlation may be attributed to the presence of the additional non-OI etiology of dental issues in the Ukrainian cohort (Lin et al., 2009; Amor et al., 2011; Lindahl et al., 2015).

Although there are numerous different genes associated with OI, the differences in phenotypes between collagen and non-collagen OI cases were insignificant, except that of blue sclera (which was also associated with collagen I pathogenic variants in previous studies) (Lindahl et al., 2015). The overlap of OI phenotypes with different genotypes remains a focus for future research.

The current study’s results corroborate the absence of a correlation between collagen pathogenic variants and hearing loss. No previous studies have detected factors, which could explain hearing loss in OI families, and this remains a vital topic for future research. Number of pediatric patients with hearing loss in Ukrainian OI cohort was high, so it would be especially interesting to identify non-collagen OI genes in remained patients (Hartikka et al., 2004; Amor et al., 2011).

## Conclusions

The current paper presents the phenotype and genotype characteristics of 94 Ukrainian OI families for the first time. These patients exhibited OI phenotypes I (46.85%), III (16.78%), IV (34.27%), and V (2.10%). *COL1A1/2* pathogenic variants were identified in 63.83% of the 94 screened unrelated patients, which were equally divided between structural (49.21%) and haploinsufficiency (50.79%) pathogenic variants. 27 novel OI causing pathogenic variants in the *COL1A1/2* genes were presented in this study. Genotype-phenotype analysis supported previous findings on the dependence of OI phenotype severity on type of defect. Future research will focus on the performance of a whole exome sequencing analysis of patients negative for *COL1A1/2* pathogenic variants, in order to identify OI genetic causes.

There appears to be very little data available on OI patients from East Slavic populations. The results of this current research will enrich the OI variant database and contribute to the understanding of genotype-phenotype correlations in osteogenesis imperfecta. The results of this research may also be used to promote further research, treatment, and diagnostics of OI in Ukraine, which will result in the improvement of patients’ quality of life and accessibility to treatment.

## Ethics Statement

The current study was conducted in accordance with the Helsinki Declaration and received approval from the Sytenko Institute of Spine and Joint Pathology of the Ukrainian Academy of Medical Sciences and the Ethical Review Committee on Human Research of the University of Tartu (Permit no. 221/M-34). Informed written consent from the patients or their legal representatives was obtained prior to inclusion to the study.

## Author Contributions

LZ conceived the study, carried out the genetic studies, interacted with the patients, performed the data analysis, participated in the design of the study, and drafted the manuscript. KM participated in the design of the study, interacted with the patients, coordinated the blood sample collection, interacted with the patients, performed the data analysis, participated in the design of the study, performed analysis and helped to draft the manuscript. AP and SKh interacted with the patients and participated in the designing of the study and sample collection. EP, SKõ and ER carried out the genetic studies, performed the data analysis, and helped to draft the manuscript. AM participated in the designing of the study, coordinated the data interpretation and statistical analysis, and helped to draft the manuscript. All authors read and approved the final manuscript.

## Funding

This study was supported by the Estonian Science Agency project IUT20-46 (TARBS14046I), the European Regional Development Fund and the Archimedes Foundation support for the Centre of Excellence on Translational Medicine, the University of Tartu’s Development Fund, University of Tartu’s Baseline Funding, and the HypOrth Project funded by the European Union’s 7^th^ Framework Programme grant agreement no. 602398.

## Conflict of Interest Statement

The authors declare that the research was conducted in the absence of any commercial or financial relationships that could be construed as a potential conflict of interest.

## Abbreviations

DI, dentinogenesis imperfecta; OI, osteogenesis imperfecta.
